# Purely in-plane ferroelectricity in monolayer SnS at room temperature

**DOI:** 10.1038/s41467-020-16291-9

**Published:** 2020-05-15

**Authors:** Naoki Higashitarumizu, Hayami Kawamoto, Chien-Ju Lee, Bo-Han Lin, Fu-Hsien Chu, Itsuki Yonemori, Tomonori Nishimura, Katsunori Wakabayashi, Wen-Hao Chang, Kosuke Nagashio

**Affiliations:** 1 Department of Materials Engineering, The University of Tokyo, 7-3-1 Hongo, Bunkyo, Tokyo 113-8656 Japan; 2 Department of Electrophysics, National Chiao Tung University, Hsinchu, 30010 Taiwan; 3 Department of Nanotechnology for Sustainable Energy, School of Science and Technology, Kwansei Gakuin University, Gakuen 2-1, Sanda, Hyogo 669-1337 Japan; 4 Center for Emergent Functional Matter Science (CEFMS), National Chiao Tung University, Hsinchu, 30010 Taiwan

**Keywords:** Electrical and electronic engineering, Condensed-matter physics, Materials for devices, Nanoscale materials, Electronic devices

## Abstract

2D van der Waals ferroelectrics have emerged as an attractive building block with immense potential to provide multifunctionality in nanoelectronics. Although several accomplishments have been reported in ferroelectric switching for out-of-plane ferroelectrics down to the monolayer, a purely in-plane ferroelectric has not been experimentally validated at the monolayer thickness. Herein, an in-plane ferroelectricity is demonstrated for micrometer-size monolayer SnS at room temperature. SnS has been commonly regarded to exhibit the odd–even effect, where the centrosymmetry breaks only in the odd-number layers to exhibit ferroelectricity. Remarkably, however, a robust room temperature ferroelectricity exists in SnS below a critical thickness of 15 layers with both an odd and even number of layers, suggesting the possibility of controlling the stacking sequence of multilayer SnS beyond the limit of ferroelectricity in the monolayer. This work will pave the way for nanoscale ferroelectric applications based on SnS as a platform for in-plane ferroelectrics.

## Abstract (web summary)

Out-of-plane ferroelectricity is usually unstable in the two dimensional limit due to the presence of the depolarization field. Here, the authors successfully circumvent this issue by growing µm-sized SnS monolayers that exhibit in-plane ferroelectricity that is stable at room temperature.

## Introduction

Nanoscale ferroelectrics have been explored for decades in areas such as nonvolatile memories, sensors, and nonlinear optoelectronics. For 3D ferroelectrics, only a few successes have been reported in downscaling the film thickness: ~1 nm BaTiO_3_^1,2^ and 1.2 nm PbTiO_3_^3^. Otherwise, ferroelectricity disappears in the nanoscale owing to the depolarization field or interfacial effects^4–6^. In contrast to the 3D materials, 2D layered materials have a dangling-bond-free surface with van der Waals (vdW) gap, and hence, they maintain the intrinsic properties even at an ultrathin thickness. Recent intensive works on 2D ferroelectrics^7–20^ have experimentally demonstrated stable ferroelectricity down to the ultimate monolayer thickness for out-of-plane ferroelectrics (MoTe_2_^7^ and WTe_2_^8^) and in-plane/out-of-plane intercorrelated ferroelectrics (*α*-In_2_Se_3_^13,14^ and SnTe^15^). Even in 2D ferroelectrics, however, the spontaneous polarization is also degraded with decreasing thickness when the 2D ferroelectric layer is vertically sandwiched with metals, owing to the depolarization field at metal/ferroelectric interfaces^21^, as in 3D ferroelectrics. Therefore, in-plane ferroelectrics are superior to the out-of-plane and intercorrelated ferroelectrics in terms of preventing the depolarization field, given that the in-plane device structure enables a large gap between the electrodes.

**Fig. 1:**
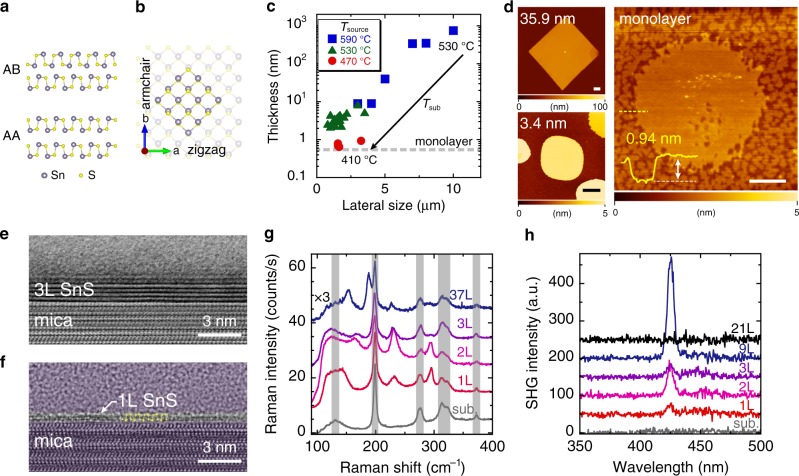
Characterization of few-to-monolayer SnS. **a** Cross-sectional crystal structures of SnS along the armchair direction with different stacking sequences: non-centrosymmetric AA and centrosymmetric AB staking. **b** Top view of crystal structure of monolayer SnS, whose twofold axis is along the armchair direction. Highlighted area shows thermodynamically stable facets. **c** The minimum thickness versus lateral size of PVD grown SnS with changing *T*_source_ and *T*_sub_. **d** AFM topographic images of SnS crystals with different thicknesses from bulk to monolayer. The scale bars represent 1 µm. **e** Cross-sectional bright-field STEM image of trilayer SnS. **f** Cross-sectional TEM image of monolayer SnS along the armchair direction. As guide to the eye, all of the region except the SnS crystal is shaded, and the atomic model is overlaid on the TEM image. **g** Thickness dependence of Raman spectrum for SnS at 3 K. The peaks in the hatch come from the mica substrate. **h** SHG spectra for SnS with different thicknesses from bulk to monolayer at RT.

The orthorhombic group-IV monochalcogenide (MX; M = Sn/Ge and X = S/Se), a purely in-plane 2D ferroelectric, has attracted considerable interest^22–27^ because ferroelasticity and ferroelectricity have been predicted as multiferroicity with a larger spontaneous polarization (*P*_s_ = 1.81–4.84 × 10^−10^ Cm^−1^)^22,23^, compared with the above-mentioned 2D ferroelectrics. Moreover, the existence of spontaneous polarization guarantees piezoelectricity, and a remarkable piezoelectric coefficient of *d* ~ 75–251 pmV^−1^ has also been predicted^28^, which is much larger than that of MoS_2_ (*d* ~ 4 pmV^−1^)^29^ and comparable to that of Pb(Zr_*x*_Ti_1−*x*_)O_3_ (*d* ~ 300 pmV^−1^). These properties dominantly originate from a puckered structure along the armchair direction as a strong anisotropy analog of black phosphorus. Given that MX is not a typical insulator for 3D ferroelectrics but a semiconductor, these properties will provide multifunctionalities in nanoscale devices. Among the MXs, SnS is the best because SnX is more chemically stable than GeX^30,31^ and the Curie temperature of SnS is higher than that of SnSe^27^. Very recently, Bao et al. reported a ferroelectric device of bulk SnS (~15 nm), breaking the centrosymmetry by applying an external electric field^32^. As the few-to-monolayer SnS has been investigated only by piezoresponse force microscopy owing to its small size of several tens of nanometers, the demonstration of a ferroelectric device for monolayer SnS has been challenging. Ferroelectricity in SnS has the odd–even effect owing to the stacking sequence of the centrosymmetric AB staking, as shown in Fig. 1a. The centrosymmetry exists in the even-number layers so that ferroelectricity is expected only in the odd-number layers and becomes prominent in few-to-monolayer SnS^22,26,32^. However, the synthesis of a high-quality monolayer SnS in the micrometer-size scale suitable for device fabrication has not been achieved^33–35^, because the interlayer interaction is strong due to the lone pair electrons in the Sn atoms, which generate a large electron distribution and electronic coupling between adjacent layers^36,37^.

Here, we report an in-plane ferroelectric device of a micrometer-size monolayer SnS grown by physical vapor deposition (PVD), where the growth conditions are precisely controlled to balance the adsorption/desorption of SnS. The Raman spectrum for monolayer SnS indicates high crystalline quality and strong anisotropy. Second harmonic generation (SHG) spectroscopy reveals that, unlike bulk SnS, monolayer SnS is non-centrosymmetric. Ferroelectric switching is successfully demonstrated for the monolayer device at room temperature (RT). Remarkably, for thin SnS below a critical thickness (~15 layers, L), the SHG signal and ferroelectric switching are also observed in the even-number SnS, thus overcoming the odd–even effect, which suggests that ultrathin SnS is grown in an unusual stacking sequence lacking centrosymmetry.

## Results

### Growth of few-to-monolayer SnS

SnS has the puckered structure along the armchair direction distorted from the NaCl structure (Fig. 1a, b), leaving the lone pair electrons in the Sn atoms. The lone pair electrons contribute to the strong interlayer force^36,37^. Therefore, the in situ observation of SnS growth has confirmed a very high growth rate in the perpendicular direction^38^. Thus, monolayer SnS has been realized by molecular beam epitaxy growth, although only with a limited crystal size of several tens of nanometers^32^. Otherwise, the minimum thickness was 5.5 nm via PVD growth^34^. To suppress the perpendicular growth rate, SnS desorption during the PVD growth was precisely controlled with growth pressure and temperature (Supplementary Fig. 1). Consequently, a thickness controllable PVD process was realized from bulk to monolayer thickness (Fig. 1c and Supplementary Fig. 2). Figure 1d and Supplementary Fig. 3 show atomic force microscopy (AFM) topographic images of SnS grown on mica substrates. For bulk SnS thicker than ~16 nm, the SnS crystal has a sharply defined diamond shape that reflects the thermodynamically stable crystal facets (Fig. 1b). With decreasing temperatures of the SnS source powder (*T*_source_) and substrate (*T*_sub_), the SnS thickness decreased and the corner became rounded. The typical temperatures (*T*_source_ and *T*_sub_) for the SnS crystals with thicknesses of ~36 nm and ~3–4 nm were (590 °C, 530 °C) and (530 °C, 410 °C), respectively. Finally, at *T*_source_ = 470 °C and *T*_sub_ = 410 °C, monolayer-thick SnS was realized with micrometer size of up to ~5 µm, which is a reasonable size for the device fabrication. Note that monolayer-thick SnS has an atomically flat surface without wedding cake morphology owing to the Stranski–Krastanov growth mode^32^ or spiral growth assisted by a screw dislocation^38^. The rounded shape could be caused by the SnS desorption during the growth and an insufficient growth time to reach the thermodynamic equilibrium state for thin SnS^35,38^. The small “holes” found in the AFM image of monolayer-thick SnS are probably etch pits created during the growth. When a post-growth annealing was performed for the SnS crystals, aligned square-shaped etch pits were created, indicating a single crystalline nature (Supplementary Fig. 4). Moreover, a lattice matching of SnS with the mica substrate was detected by the in-plane X-ray diffraction (XRD) measurement, suggesting a strong SnS/mica interaction (Supplementary Fig. 5)^39^.

### Characterization of few-to-monolayer SnS

Figure 1e shows a cross-sectional bright-field scanning transmission electron microscopy (STEM) image of trilayer SnS. For trilayer SnS, the composition ratio Sn:S was 1:0.8 from energy-dispersive X-ray spectroscopy (Supplementary Fig. 6), indicating the absence of other S rich phases (e.g., SnS_2_ and Sn_2_S_3_). Interestingly, from the TEM image of trilayer SnS, the monolayer thickness *d*_1L_ was determined to be 5.8 Å, larger than that of the mechanically exfoliated bulk sample (*d*_1L_ ~ 5.4 Å^37^). This expansion suggests the possibility of an unusual stacking sequence rather than the AB stacking. From an ab initio simulation, the AA stacked bilayer SnS (Fig. 1a) indicates *d*_1L_ = 6.34 Å, larger than *d*_1L_ = 5.85 Å for the AB stacking. For the TEM observation of monolayer SnS, the crystal orientation was determined beforehand by polarized Raman spectroscopy, as discussed later, because the TEM image of monolayer SnS is much more indistinct than that of the bulk crystals owing to the degradation during the sample preparation by the focused ion beam process and the TEM observation itself. By adjusting the zone axis, it can be observed that two sub-layers have a monolayer structure, which matches well with the configuration along the armchair direction (Fig. 1f).

Figure 1g shows Raman spectra for PVD grown SnS with different thicknesses from bulk to monolayer, measured at 3 K. For a bulk SnS (~37 L), specific peaks were observed at 153.7, 188.3, 227.6, and 291.8 cm^−1^ in addition to the peaks from mica substrate at ~131, 198.9, 276.0, 312.8, 322.9, and 372.5 cm^−1^. These peak positions are well consistent with those of bulk SnS via mechanical exfoliation or PVD^33–35,37^. With a decreased thickness from trilayer to monolayer, the Raman peak positions at ~230 and 293 cm^−1^ almost coincided with each other, while those between 140 and 190 cm^−1^ changed significantly. For monolayer SnS, a Raman peak was observed at ~145 cm^−1^, and also at 232.4 and 295.0 cm^−1^. A similar trend was also obtained at RT (Supplementary Fig. 7). Those values will be discussed later with the results of polarized Raman spectroscopy and the phonon mode calculation. To determine the non-centrosymmetry, which is required for ferroelectricity, µ-SHG spectroscopy^40,41^ was carried out for different thicknesses, from bulk to monolayer (Fig. 1h). An 850-nm laser was used as the excitation source. The bulk SnS, thicker than ~21 L, showed no SHG signal, while SnS under the critical thickness of ~15 L showed SHG signal at *λ* = 425 nm. Although the odd–even effect was expected for the AB stacked SnS, it was found that all the ultrathin SnS flakes, including the even-number layers, showed SHG signals below the critical thickness (Supplementary Fig. 8). For confirmation, mica substrate was measured under the same conditions. Although it is known that a weak SHG signal can be generated even for the centrosymmetric material due to the surface SHG effect^40^, no SHG signal was detected from the mica surface (Fig. 1h). These results suggest an unusual stacking sequence of the PVD grown SnS, along with the results of interlayer distance from the TEM image.

### Anisotropic characteristics for monolayer SnS

**Fig. 2:**
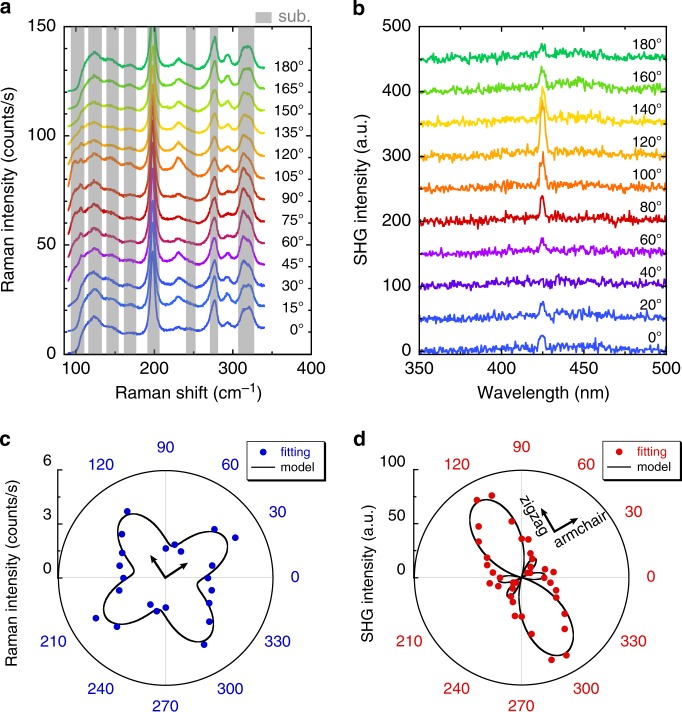
Optical anisotropies of monolayer SnS. **a**, **b** Polarization dependences of Raman (3 K) and SHG (RT) spectrum of monolayer SnS, with parallel and perpendicular polarization, respectively. The gray shaded region of Raman spectra represents Raman peak from mica substrate. **c**, **d** Polar plots of Raman intensity at ~234.0 cm^−1^ and SHG intensity at 425 nm, respectively. The inset axes show the armchair and zigzag directions.

**Fig. 3:**
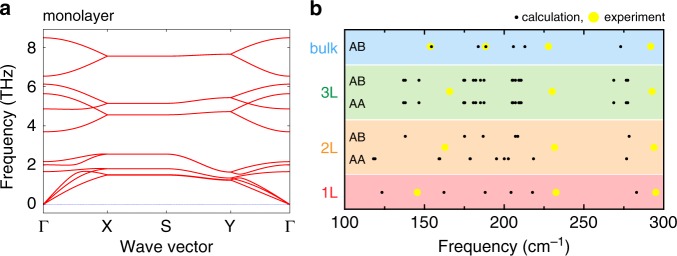
Theoretical calculation of phonon dispersion and Raman active modes. **a** Phonon dispersion of the monolayer SnS. **b** Comparison of calculated Raman active modes and experimental Raman peak positions for SnS with different thicknesses (monolayer, bilayer, trilayer, and bulk) and stacking sequences (AA and AB).

In monolayer SnS, the dipole moment along the armchair direction leads to spontaneous polarization^22,23^. Different from the thick SnS, it is difficult to identify the orientation of the present few-to-monolayer crystals from their shape, as mentioned above. To characterize the in-plane anisotropy, the angular dependences of both Raman and SHG have been investigated. Figure 2a shows the polarization dependence of the Raman spectrum for monolayer SnS under the parallel polarization configuration at 3 K (Supplementary Fig. 9). Specific peaks were observed at ~234 and 294 cm^−1^, which are consistent with the results of the unpolarized Raman measurement, although it was difficult to determine the precise peak position between 100 and 200 cm^−1^ because of the overlaps with peaks from the substrate. To investigate the relationship between the Raman active modes and stacking sequences for SnS, an ab initio calculation was carried out using the Vienna ab initio simulation package (VASP)^42^. Figure 3a shows a typical example of phonon dispersion along the path passing through the main high-symmetry *k*-points in the irreducible Brillouin zone of monolayer SnS. Figure 3b and Supplementary Fig. 10 summarize the Raman active phonon modes for the AB and AA stacked SnS with different number of layers. For bulk SnS, the experimental results almost agree with the calculated results, whereas there are large differences between experiments and calculation for the few-to-monolayer SnS. The origin of this difference is probably the strain incorporated through the interaction with the mica substrate, as discussed above. By comparing the calculated Raman active modes and experimental results, the Raman signals of the monolayer at 234 cm^−1^ is attributed to the *A*_1_ mode of the *C*_2v_ point group (Supplementary Fig. 10). As expected, the Raman peak intensity at 234 cm^−1^ shows a significant change as a function of the rotation angle (Fig. 2a). The Raman tensor *R* for the *A*_1_ mode of *C*_2v_ point group can be written as ref. ^43^

1 $$R\left( {A_1} \right) = \left( {\begin{array}{*{20}{c}} {|A|e^{{\mathrm{i}}\varphi _A}} & 0 & 0 \\ 0 & {|B|e^{{\mathrm{i}}\varphi _B}} & 0 \\ 0 & 0 & {|C|e^{{\mathrm{i}}\varphi _C}} \end{array}} \right),$$

which is the same as the *A*_g_ mode of bulk SnS (*D*_2h_ point group) that shows a strong anisotropy^33–35^. The unitary vector of incident light is **e**_i_ = (cos *θ*, sin *θ*, 0), where *θ* is the polarization angle defined as the angle between the incident light and the zigzag direction of SnS crystal. The unitary vector of scattered light is **e**_s_ = (cos *θ*, sin *θ*, 0) and (−sin *θ*, cos *θ*, 0) for the parallel (‖) and perpendicular (⊥) polarization, respectively. For the polarized Raman intensity of the *A*_1_ peak, the angular dependences can be calculated using the following equations^35^:

2 $$I_\parallel \propto \left| A \right|^2{\mathrm{cos}}^{4} \, \theta + \left| B \right|^2{\mathrm{sin}}^4 \, \theta + 2\left| A \right|\left| B \right|{\mathrm{cos}}^{2} \, \theta \, {\mathrm{sin}}^{2} \, \theta \, {\mathrm{cos}} \, \varphi _{BA},$$

3 $$I_ \bot \propto \frac{{\left| A \right|^2 + \left| B \right|^2 - 2\left| A \right|\left| B \right|{\mathrm{cos}} \, \varphi _{BA}}}{4}{\mathrm{sin}}^2 \, 2\theta,$$

where *φ*_*BA*_ = |*φ*_B_ – *φ*_A_| is the phase difference between the Raman tensor elements $\left| A \right|e^{{\mathrm{i}}\varphi _A}$ and $\left| B \right|e^{{\mathrm{i}}\varphi _B}$. By fitting the experimental data with Eq. (2), the crystal orientation was revealed (Fig. 2c). As in the polarized Raman spectroscopy, Fig. 2b shows a strong angular dependence of SHG for monolayer SnS with perpendicular polarization configuration. For the polarized SHG intensity under parallel and perpendicular polarization, the angular dependence in the *C*_2v_ point group is written as ref. ^44^

4 $$\chi _\parallel ^{(2)} = \left( {\chi _{xyx}^{(2)} + \chi _{yxx}^{(2)}} \right){\mathrm{sin}} \, \theta \, {\mathrm{cos}}^{2} \, \theta + \chi _{yyy}^{(2)}\,{\mathrm{sin}}^{3} \, \theta,$$

5 $$\chi _ \bot ^{(2)} = \chi _{yxx}^{(2)}{\mathrm{cos}}^3 \, \theta + \left( {\chi _{yyy}^{(2)} - \chi _{xyx}^{(2)}} \right){\mathrm{cos}} \, \theta \, {\mathrm{sin}}^{2} \, \theta,$$

where $\chi _{ijk}^{(2)}$ is the SHG susceptibility tensor element along the different directions. We fitted the experimental data based on Eq. (5) to determine the zigzag/armchair orientation, as shown in Fig. 2d. The measured patterns agree well with the theoretical model. The anisotropy revealed from the polarized Raman and SHG spectra indicates again the high crystallinity of monolayer SnS.

### Modulation of Schottky barrier height at metal/SnS interface

**Fig. 4:**
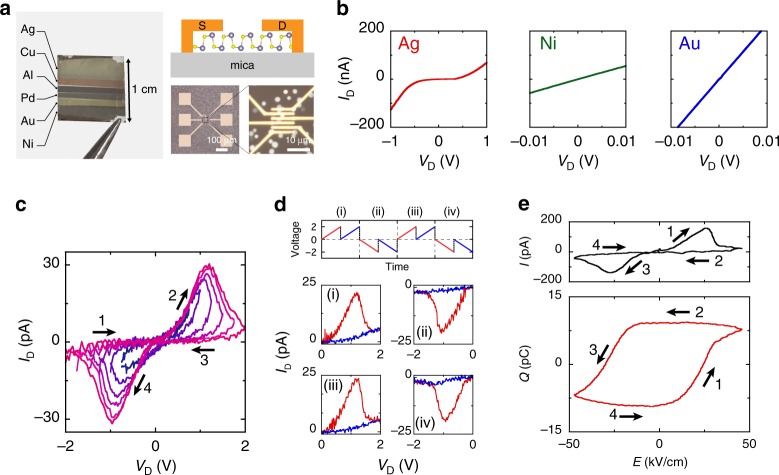
Ferroelectric switching behaviors of few-layer SnS. **a** Left: Photograph of mica substrate after the deposition of various metals with different work functions. Right: Cross-sectional schematic and optical images of two-terminal SnS devices. Typical channel length *l*_ch_ and width *w*_ch_ were *l*_ch_ = 0.4–0.8 µm and *w*_ch_ = 3–5 µm, respectively. **b** *I*_D_–*V*_D_ curves for bulk SnS with different metal contacts: Ag, Ni, and Au. **c** RT *I*_D_–*V*_D_ for Ag/9L-SnS device with different *V*_D_ sweep ranges. *V*_D_ was swept from minus to plus to minus (e.g., −1 → +1 → −1 V). **d** Double-wave measurement from 0 to 2 and 0 to −2 V. Top: applied voltage along time. The voltage was applied two times at the positive and negative bias repeatedly. Bottom: *I*_D_–*V*_D_ curves for different sweeps (i)–(iv). The red and blue lines represent the first and second sweep, respectively. **e** Ferroelectric resistive switching for Ag/SnS: current and charge versus nominal electric field measured by ferroelectric measurement system at 1 Hz and RT.

Switching of the spontaneous polarization and SHG is required to prove the ferroelectricity in SnS. A tip poling experiment by using scanning probe microscopy is an effective way to observe the polarization switching for the local area. However, it is more difficult to detect the trace of spontaneous polarization in SnS than that in out-of-plane ferroelectrics because in-plane ferroelectricity does not respond to the out-of-plane electric field applied by the probe tip. To demonstrate the in-plane polarization switching, in-plane two-terminal devices with source/drain electrodes on SnS crystals were fabricated (Fig. 4a and Supplementary Fig. 12). During the fabrication process, the exact locations of SnS crystals were captured by optical images so that only one crystal was contacted by two adjacent electrodes. Note again that SnS is a semiconductor with energy gaps of 1.5 eV for monolayer and 1.1 eV for bulk^45^, where channel conductance makes it difficult to identify the very small displacement current^46^. To prevent the channel conductance by forming an insulator-like interface, the Schottky barrier height (SBH) has been investigated by changing the metal work function (*Φ*_m_). After bulk SnS (~20 nm) was grown on the mica substrate, a standard electron beam lithography was performed followed by multiple metal depositions with a series of metals (In, Al, Ag, Cu, Ni, Pd, and Au), as shown in Fig. 4a. Assuming that the metal/SnS interface is ideal and free from Fermi level pinning^47^, the SBH will strongly depend on *Φ*_m_. In such a case, the metal with smaller *Φ*_m_ is preferable to increase the SBH for the *p*-type semiconductor SnS^33,37,48,49^. At RT, ohmic *I*_D_–*V*_D_ curves were obtained for bulk SnS with metals of In, Cu, Ni, Pd, and Au, while Schottky *I*_D_–*V*_D_ with Al and Ag (Fig. 4b, and Supplementary Fig. 13).

### Ferroelectric switching device of few-to-monolayer SnS

Figure 4c shows *I*_D_–*V*_D_ curves for 9 L SnS with the Ag contact measured by increasing the *V*_D_ sweep range at RT. Unlike the *I*_D_–*V*_D_ curve without hysteresis for the ohmic Ni metal contact (Supplementary Fig. 14), the Schottky Ag contact exhibited a well-reproducible hysteresis (Supplementary Fig. 15). Although it is known that Ag could give memristor behavior owing to its high diffusivity^50,51^, no significant Ag diffusion was confirmed from the AFM images at the low resistive state (Supplementary Fig. 16). Moreover, the larger drain bias led to a larger window of hysteresis loop, distinguishing a maximum conductivity at approximately *V*_D_ = ±1 V. To confirm that this hysteresis originates from the ferroelectric switching, a double-wave measurement was performed^52^. When *V*_D_ was applied from 0 to 2 V for two times, a current peak was observed only in the first sweep and it turned to be a highly resistive state in the second sweep (Fig. 4d). Similarly, a negative *V*_D_ sweep (0 to −2 V) showed a current peak only once in the first sweep. This is because polar switching results in the current peak in the first sweep, but never in the second sweep because the direction of polarization is steady. Therefore, this result of double-wave measurement is strong evidence for the switching of spontaneous polarization, that is, ferroelectricity. It should be emphasized that the crystal orientation of SnS was not determined before the electrode fabrication. Despite the fact that spontaneous polarization exists along the armchair direction, the *I*_D_–*V*_D_ hysteresis loop was observed regardless of the initial crystal orientation that the external electric field is applied. This result suggests that the more flexible polarization switching due to the possible existence of the domain structure. Furthermore, even an external electric field along the non-ferroelectric zigzag direction could induce ferroelectricity with structural rearrangement into the armchair structure^53^ in a similar way as ferroelasticity^22,23^.

To further analyze the switching behavior quantitatively, *Q*–*E* (*Q* is charge, and *E* is electric field) curve was measured using a ferroelectric evaluation system. When the AC bias at 1 Hz was applied (0 → +2 → −2 → 0 V), an *I*–*E* curve drew a hysteresis loop very similar to the *I*_D_–*V*_D_ curve under DC bias (Fig. 4e). Here, *E* is assumed to be *V*_D_/*l*_ch_, where *l*_ch_ is the channel length, which should overestimate *E* because there is also a voltage drop at the Schottky contact. The *Q*–*E* loop corresponding to the *I*–*E* loop is shown at the bottom of Fig. 4e. A distinct hysteresis loop was obtained with the charge of ~9 pC, which is a characteristic of ferroelectrics in general when the external electric field switches the polarization to generate the displacement current. However, the remnant polarization *P*_*r*_ = *Q*/*w*_ch_, where *w*_ch_ is channel width, was determined to be *P*_*r*_ ~ 3 µCm^−1^, which is almost four orders of magnitude larger than the theoretical value of *P*_*r*_ = 260 pCm^−1^^23^. This discrepancy suggests that the hysteresis loop is not mainly due to the displacement current. The hysteresis loop can be dominantly caused by the contribution of other factors in addition to the displacement current, as discussed below.

Considering it has been suggestively pointed out that the closed *Q*–*E* loop can be artificially obtained even for non-ferroelectric materials when they are lossy or leaky^54,55^, the large discrepancy on the remnant polarization for SnS is carefully discussed as follows. One possible case is for films with a large concentration of traps near the metal/film interface^55^. The number of traps required to reproduce the *Q*–*E* loop in Fig. 4e was estimated. As it corresponds to one in three of the total number of atoms in the SnS channel material, the traps are not the origin for the large *P* (Supplementary Fig. 17). The other case is the resistive switching^56^, where a hysteresis loop similar to that in Fig. 4e has been discussed as an effect of SBH modulation at the metal/ferroelectric interface owing to the reversed polarization for the previous works on ferroelectric 2D materials (MoTe_2_^7^, WTe_2_^8^, *α*-In_2_Se_3_^13,14,18,19^, SnTe^15^, and CuInP_2_S_6_^16,17^). In the present study, the current flowing through the SnS channel was drastically suppressed by selecting the strong Schottky contact metal. Nevertheless, the present ferroelectric *Q*–*E* hysteresis loop is considered to be owing to the resistive change at the metal/SnS Schottky interface accompanied with polar switching because the displacement current level for in-plane 2D devices is negligibly small. Moreover, the coercive electric field *E*_c_, which originates this switching, was found to be ~25 kV cm^−1^ for 9 L SnS. This value is comparable to the experimental value for bulk SnS with gate-induced non-centrosymmetry (~10.7 kVcm^−1^)^32^, whereas it is much smaller than the theoretical value for monolayer (1.8 × 10^3^ kV cm^−1^)^24^. This discrepancy between the experiment and calculation is probably related to the existence of a mobile domain wall or lattice strain in SnS caused by the difference in thermal expansion coefficients between SnS and the mica substrate in the real system^23^.

## Discussion

**Fig. 5:**
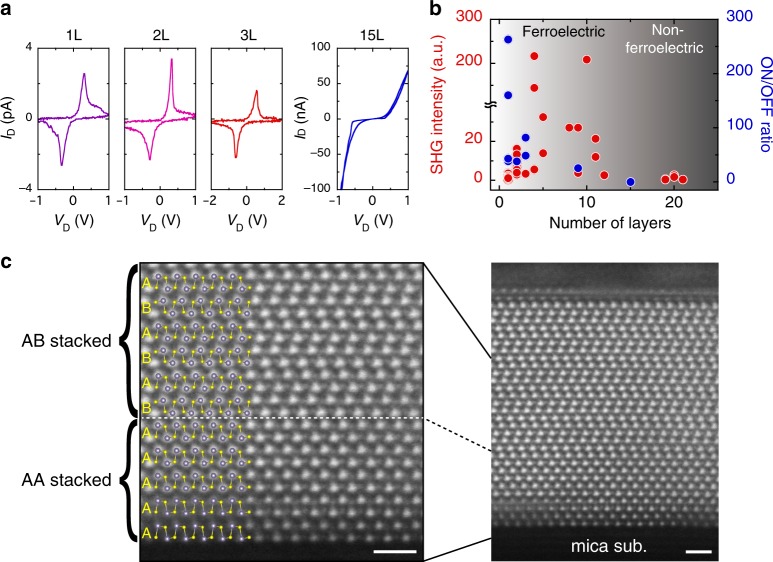
Transition of stacking sequence from AA to AB staking. **a** Ferroelectric resistive switching for SnS with different thicknesses: monolayer, bilayer, trilayer, and 15 L. **b** Thickness dependences of the SHG intensity and ON/OFF ratio for different thicknesses. The ON/OFF ratio was determined at the coercive electric field of *I*_D_–*V*_D_ for Ag/SnS device. Each data points of SHG and ON/OFF ratio represent an individual SnS crystal. **c** Cross-sectional HAADF-STEM image of 16 L SnS along the armchair direction. The scale bars represent 1 nm.

For further understanding the dependences of SHG and ferroelectric switching on the number of layers, the SnS thickness was systematically changed. Figure 5a shows *I*_D_–*V*_D_ curves measured in the same way as that in Fig. 4c. For the monolayer, bilayer, and trilayer, the ferroelectric switching was realized as in 9 L SnS, as discussed above. For SnS thicker than 15 L, the current leakage through the SnS channel dramatically increased and it was difficult to observe the ferroelectric hysteresis loop. To quantify the effect of polar switching on the resistivity, the conductivity ratio for the low resistive state (ON) and high resistive state (OFF) at *E*_c_ was calculated. Figure 5b shows the dependence of the ON/OFF ratio on the number of layers together with the SHG intensity. A large distribution was found in the SHG intensity for each number of layers, which is probably caused by the variation of crystalline quality due to the desorption-controlled PVD growth. This heterogeneity was also found when a different batch of SnS powder source was used. That is, the maximum SHG intensity for SnS grown via one batch exceeded that grown via the other batch. This result suggests the possibility of further increase in the SnS crystalline quality with the improvement of the SnS powder source. Despite of these dispersions, the SHG intensity tends to increase with a number of layers up to ~10 L, then it decreases, and finally quenches above 21 L. The ferroelectric switching only occurs below this critical thickness. These results indicate that there is a change in the stacking sequence from the AA to AB stacking, which determines the existence of ferroelectricity (Supplementary Fig. 19). In order to prove the stacking transition, cross-sectional structure was further investigated for 16 L SnS by TEM observation. For this sample, the crystal orientation was determined from the diamond-shaped-like crystal structure so that we can observe the atomic configuration along the armchair direction, where AA and AB stackings can be identified (Fig. 5c and Supplementary Fig. 20). Figure 5c shows high-angle annular dark-field (HAADF) STEM image of 16 L SnS along the armchair direction. The stacking transition from AA to AB is clearly observed at the thickness of 6 L. Even though this transition plane was steeply continuous through a selected region of several tens of nanometers, it can be possible that the transitions occur at different thicknesses in a micrometer scale and from sample to sample, resulting in the discrepancy between critical thicknesses determined from TEM and SHG measurements. The unusual growth mode is probably due to the substrate effect, such as lattice strain and electrostatic surface charges. In the previous work on bulk SnS (~15 nm)^32^, an *I*_D_–*V*_D_ hysteresis loop similar to that in the present work was observed for in-plane two-terminal Au contact devices, where the ferroelectricity in bulk SnS was achieved by extrinsically breaking the inversion symmetry through the perpendicular electric field from the back gate. It should be emphasized that the intrinsic ferroelectricity is observed for monolayer SnS in this study.

In conclusion, monolayer SnS with micrometer size is grown by precisely controlling the growth pressure and temperature in PVD. The lack of the centrosymmetry and strong anisotropy rooted from the puckered structure are confirmed based on polarized Raman and SHG spectroscopies. After the current flowing through the SnS channel in two-terminal devices was suppressed by selecting the strong Schottky Ag contact, RT in-plane ferroelectric switching was realized by the double-wave method. Remarkably, the robust RT ferroelectricity was identified in SnS below the critical thickness of ~15 L, probably due to the interaction with the substrate. This result suggests a possibility of controlling the stacking sequence of multilayer SnS, going beyond the limit of ferroelectricity in monolayer SnS. Given that SnS is the semiconductor with multiferroicity^22,23^, innately exhibiting pyroelectricity and piezoelectricity, this work will open up possibilities of providing multifunctionalities in vdW heterostructure devices.

## Methods

### PVD growth

SnS crystals were grown by a home-built PVD growth furnace with three heating zones (Supplementary Fig. 1). A commercially available SnS powder was used as a source. To promote lateral growth, we used a freshly cleaved mica substrate sized 1 cm × 1 cm × 0.5 mm, whose surface is atomically flat. N_2_ carrier gas was introduced into the furnace through the mass flow controller and the growth pressure was reduced to 10 Pa by a vacuum system to enhance the SnS desorption during the growth.

### Optical characterizations

µ-Raman spectra were measured using a 488 nm excitation laser, whose penetration depth is ~20 nm in SnS. The nominal 1/e^2^ spot diameter and laser power on the sample surface were 2.5 µm and 0.5 mW, respectively. To avoid degradation of SnS during the measurement, the samples were measured in the vacuum. The SHG measurements were conducted using a mode-locking Ti:sapphire laser (wavelength: 850 nm, pulse width: ~150 fs and repetition rate: 80 MHz) in a home-built optical microscope under the backscattering configuration. The laser pulse was focused to a spot size ~1.1 μm on the sample by a 100× objective lens. The backscattered SHG signals were sent into a 0.75-m monochromator and detected by a nitrogen-cooled CCD camera. For polarization-resolved SHG, the sample was mounted on a motorized rotational stage. The linear polarization of the excitation laser and SHG signals was selected and analyzed separately by polarizers and half-wave plates.

### Ab initio simulation

We have used the VASP to perform first-principle calculations based on density functional theory to study geometric and electric properties^42^. The exchange and correlation potentials are the Perdew–Burke–Ernzerhof functional and is treated using the generalized gradient approximation^57^. We employed the Monkhorst-Pack scheme to sample reciprocal space with Γ-centered 16 × 16 × 1 grid for geometry relaxations of 2D systems and 16 × 16 × 4 grids for that of bulk system. The plane-wave basis cutoff energy is set to be 500 eV. The convergence criterion is set to be 10^−5^ eV for energy in SCF cycles. And the full relaxation is continued until the residual force is less than 0.01 eV Å^−1^. We set 25 Å vacuum perpendicular to the 2D plane is used to avoid the interaction between replaced atoms. In addition, Grimme’s DFT-D2^58^ method implemented in VASP is invoked to correct the vdW-like interaction existing in these systems. Moreover, we calculated phonon dispersion at Γ-point based on the density functional perturbation theory. In addition, the calculation of phonon dispersion and irreducible representation has been implemented using phonopy^59^.

### Device fabrication and transport characterization

Two-terminal devices were fabricated with electrode pattering using standard electrode beam lithography. Electrode metals (In, Al, Ag, Cu, Ni, Pd, and Au) were deposited via thermal evaporation, followed by additional Au deposition as passivation for the source/drain metals (Supplementary Fig. 12). The electrical transport characteristics were measured in vacuum to avoid sample degradation. The *Q*–*E* curves were measured by a ferroelectric evaluation system (FCE-1A, Toyo corporation) with samples set in vacuum.

## Supplementary information

Supplementary Information

## Data Availability

The data that support the findings of this study are available from the corresponding authors upon reasonable request.

## References

[1] Lee SR (2019). First Observation of ferroelectricity in ~1 nm ultrathin semiconducting BaTiO_3_ films. Nano Lett..

[2] Ji D (2019). Freestanding crystalline oxide perovskites down to the monolayer limit. Nature.

[3] Fong DD (2004). Ferroelectricity in ultrathin perovskite films. Science.

[4] Mehta RR, Silverman BD, Jacobs JT (1973). Depolarization fields in thin ferroelectric films. J. Appl. Phys..

[5] Junquera J, Ghosez P (2003). Critical thickness for ferroelectricity in perovskite ultrathin films. Nature.

[6] Stengel M, Spaldin NA (2006). Origin of the dielectric dead layer in nanoscale capacitors. Nature.

[7] Yuan S (2019). Room-temperature ferroelectricity in MoTe_2_ down to the atomic monolayer limit. Nat. Commun..

[8] Fei Z (2018). Ferroelectric switching of a two-dimensional metal. Nature.

[9] You L (2019). Origin of giant negative piezoelectricity in a layered van der Waals ferroelectric. Sci. Adv..

[10] Zhou Y (2017). Out-of-plane piezoelectricity and ferroelectricity in layered α-In_2_Se_3_ nanoflakes. Nano Lett..

[11] Zheng C (2018). Room temperature in-plane ferroelectricity in van der Waals In_2_Se_3_. Sci. Adv..

[12] Xiao J (2018). Intrinsic two-dimensional ferroelectricity with dipole locking. Phys. Rev. Lett..

[13] Cui C (2018). Intercorrelated in-plane and out-of-plane ferroelectricity in ultrathin two-dimensional layered semiconductor In_2_Se_3_. Nano Lett..

[14] Xue F (2018). Room-temperature ferroelectricity in hexagonally layered α-In_2_Se_3_ nanoflakes down to the monolayer limit. Adv. Funct. Mater..

[15] Chang K (2016). Discovery of robust in-plane ferroelectricity in atomic-thick SnTe. Science.

[16] Liu F (2016). Room-temperature ferroelectricity in CuInP_2_S_6_ ultrathin flakes. Nat. Commun..

[17] Si M, Liao P-Y, Qiu G, Duan Y, Ye PD (2018). Ferroelectric field-effect transistors based on MoS_2_ and CuInP_2_S_6_ two-dimensional van der Waals heterostructure. ACS Nano.

[18] Xue F (2019). Gate-tunable and multidirection-switchable memristive phenomena in a van der Waals ferroelectric. Adv. Mater..

[19] Wan S (2018). Room-temperature ferroelectricity and a switchable diode effect in two-dimensional α-In_2_Se_3_ thin layers. Nanoscale.

[20] Belianinov A (2015). CuInP_2_S_6_ room temperature layered ferroelectric. Nano Lett..

[21] Xue F (2018). Multidirection piezoelectricity in mono- and multilayered hexagonal α-In_2_Se_3_. ACS Nano.

[22] Wu M, Zeng XC (2016). Intrinsic ferroelasticity and/or multiferroicity in two-dimensional phosphorene and phosphorene analogues. Nano Lett..

[23] Wang H, Qian X (2017). Two-dimensional multiferroics in monolayer group IV monochalcogenides. 2D Mater..

[24] Hanakata PZ, Carvalho A, Campbell DK, Park HS (2016). Polarization and valley switching in monolayer group-IV monochalcogenides. Phys. Rev. B.

[25] Barraza-Lopez S, Kaloni TP, Poudel SP, Kumar P (2018). Tuning the ferroelectric-to-paraelectric transition temperature and dipole orientation of group-IV monochalcogenide monolayers. Phys. Rev. B.

[26] Lebedev AI (2018). Ferroelectricity and piezoelectricity in monolayers and nanoplatelets of SnS. J. Appl. Phys..

[27] Fei R, Kang W, Yang L (2016). Ferroelectricity and phase transitions in monolayer group-IV monochalcogenides. Phys. Rev. Lett..

[28] Fei R, Li W, Li J, Yang L (2015). Giant piezoelectricity of monolayer group IV monochalcogenides: SnSe, SnS, GeSe, and GeS. Appl. Phys. Lett..

[29] Duerloo K-AN, Ong MT, Reed EJ (2012). Intrinsic piezoelectricity in two-dimensional materials. J. Phys. Chem. Lett..

[30] Higashitarumizu N, Kawamoto H, Ueno K, Nagashio K (2018). Fabrication and surface engineering of two-dimensional SnS toward piezoelectric nanogenerator application. MRS Adv..

[31] Guo Y, Zhou S, Bai Y, Zhao J (2017). Oxidation resistance of monolayer group-IV monochalcogenides. ACS Appl. Mater. Interfaces.

[32] Bao Y (2019). Gate-tunable in-plane ferroelectricity in few-layer SnS. Nano Lett..

[33] Tian Z, Guo C, Zhao M, Li R, Xue J (2017). Two-dimensional SnS: a phosphorene analogue with strong in-plane electronic anisotropy. ACS Nano.

[34] Xia J (2016). Physical vapor deposition synthesis of two-dimensional orthorhombic SnS flakes with strong angle/temperature-dependent Raman responses. Nanoscale.

[35] Li M (2017). Revealing anisotropy and thickness dependence of Raman spectra for SnS flakes. RSC Adv..

[36] Song H-Y, Lü J-T (2018). Density functional theory study of inter-layer coupling in bulk tin selenide. Chem. Phys. Lett..

[37] Higashitarumizu N (2018). Self-passivated ultra-thin SnS layers via mechanical exfoliation and post-oxidation. Nanoscale.

[38] Sutter P, Sutter E (2018). Growth mechanisms of anisotropic layered group IV chalcogenides on van der Waals substrates for energy conversion applications. ACS Appl. Nano Mater..

[39] Wang SF, Fong WK, Wang W, Surya C (2014). Growth of highly textured SnS on mica using an SnSe buffer layer. Thin Solid Films.

[40] Li Y (2013). Probing symmetry properties of few-layer MoS_2_ and h-BN by optical second-harmonic generation. Nano Lett..

[41] Hsu W-T (2014). Second harmonic generation from artificially stacked transition metal dichalcogenide twisted bilayers. ACS Nano.

[42] Kresse G, Furthmüller J (1996). Efficient iterative schemes for ab initio total-energy calculations using a plane-wave basis set. Phys. Rev. B.

[43] Loudon R (1964). The Raman effect in crystals. Adv. Phys..

[44] Wang H, Qian X (2017). Giant optical second harmonic generation in two-dimensional multiferroics. Nano Lett..

[45] Sun Y (2014). All-surface-atomic-metal chalcogenide sheets for high-efficiency visible-light photoelectrochemical water splitting. Adv. Energy Mater..

[46] Pintilie L, Stancu V, Trupina L, Pintilie I (2010). Ferroelectric Schottky diode behavior from a SrRuO_3_-Pb(Zr0.2Ti0.8)O3-Ta structure. Phys. Rev. B.

[47] Liu Y (2018). Approaching the Schottky–Mott limit in van der Waals metal–semiconductor junctions. Nature.

[48] Vidal J (2012). Band-structure, optical properties, and defect physics of the photovoltaic semiconductor SnS. Appl. Phys. Lett..

[49] Hajzus JR (2017). Contacts to solution-synthesized SnS nanoribbons: dependence of barrier height on metal work function. Nanoscale.

[50] Longnos F (2013). On the impact of Ag doping on performance and reliability of GeS_2_-based conductive bridge memories. Solid. State Electron..

[51] Yin S (2019). Emulation of learning and memory behaviors by memristor based on Ag migration on 2D MoS_2_ surface. Phys. Status Solidi.

[52] Fukunaga M, Noda Y (2008). New technique for measuring ferroelectric and antiferroelectric hysteresis loops. J. Phys. Soc. Jpn..

[53] Shen X-W, Tong W-Y, Gong S-J, Duan C-G (2017). Electrically tunable polarizer based on 2D orthorhombic ferrovalley materials. 2D Mater..

[54] Scott JF (2008). Ferroelectrics go bananas. J. Phys. Condens. Matter.

[55] Pintilie L, Alexe M (2005). Ferroelectric-like hysteresis loop in nonferroelectric systems. Appl. Phys. Lett..

[56] Tsurumaki A, Yamada H, Sawa A (2012). Impact of Bi deficiencies on ferroelectric resistive switching characteristics observed at *p*-type Schottky-like Pt/Bi_1-δ_FeO_3_ interfaces. Adv. Funct. Mater..

[57] Perdew JP, Burke K, Ernzerhof M (1996). Generalized gradient approximation made simple. Phys. Rev. Lett..

[58] Grimme S (2006). Semiempirical GGA-type density functional constructed with a long-range dispersion correction. J. Comput. Chem..

[59] Togo A, Tanaka I (2015). First principles phonon calculations in materials science. Scr. Mater..

