# Supplementary material for: Purely in-plane ferroelectricity in monolayer SnS at room temperature
Source: Nat Commun. 2020 May 15;11:2428. doi: 10.1038/s41467-020-16291-9 (PMC7229038; doi:10.1038/s41467-020-16291-9)
Supplement: Supplementary file 1 — Supplementary Information [file 41467_2020_16291_MOESM1_ESM.pdf]

Supplementary Information

Purely in-plane ferroelectricity in monolayer SnS at room temperature

Higashitarumizu *et al*.

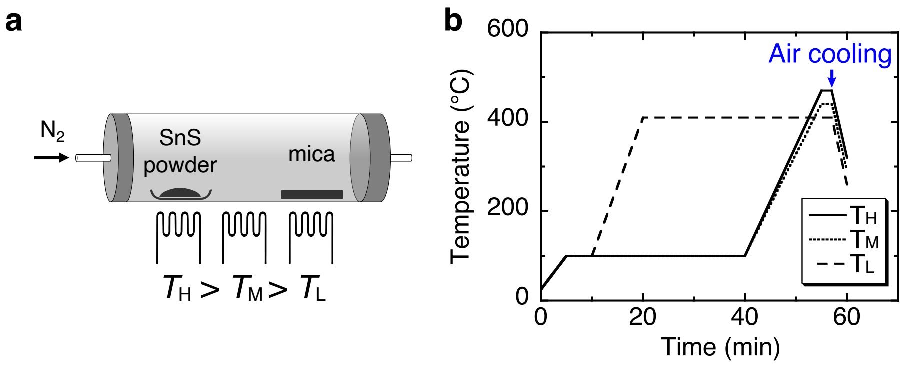

Supplementary Fig. 1 Growth conditions for monolayer SnS. **a** Schematic diagram of three-zone PVD chamber by separately controlling the heaters at high, middle, and low temperatures (T_H, T_M, and T_L) **b** Typical temperature profiles for monolayer growth. After the growth, the chamber was air-cooled at the rate of ~50 °C/min.

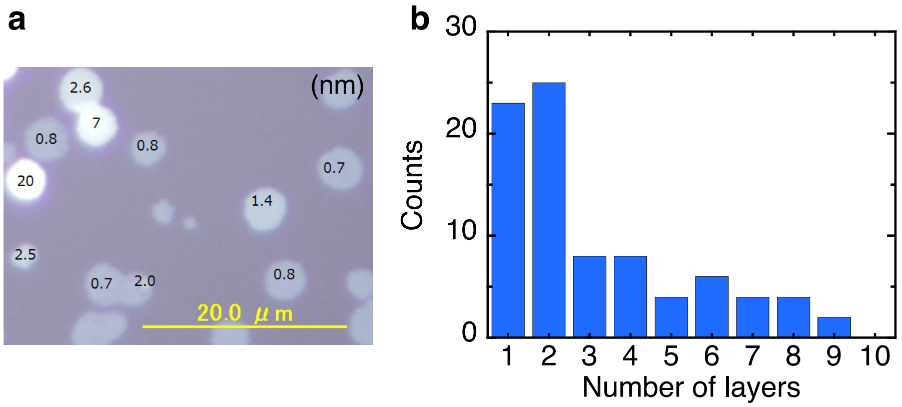

Supplementary Fig. 2 Thickness distribution of PVD grown SnS. **a** Typical optical image and **b** thickness distribution histogram of SnS on mica with different thickness obtained via the same substrate and growth conditions (Supplementary Fig. 1b).

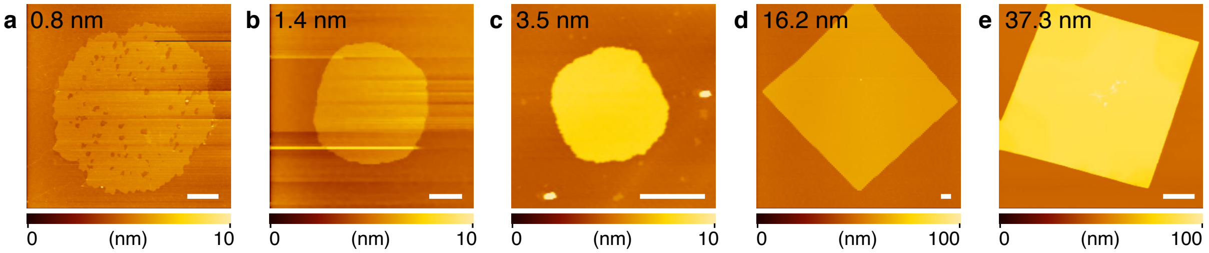

Supplementary Fig. 3 Evolution of crystal morphology for different SnS thicknesses. **a** 0.8 nm, **b** 1.4 nm, **c** 3.5 nm, **d** 16.2 nm, **e** 37.3 nm. The scale bars represent 1 μm.

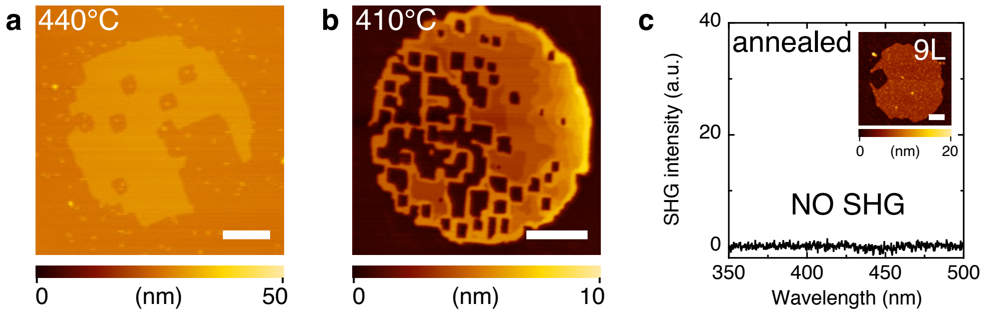

Supplementary Fig. 4 Effects of post-growth annealing on surface morphology and SHG property of SnS: AFM topographic images of multilayer SnS after post-growth annealing in N_2 atmosphere at **a** 440°C and **b** 410°C. Square-shaped etch pits were clearly observed within the SnS crystals, and their lateral size becomes larger with increasing annealing temperature. **c** SHG measurement for 9L SnS after the annealing at 410°C. As shown in the inset AFM image, a square-shaped etch pit were formed near the crystal edge. Even though no other etch pits were found in much of the SnS surface region, SHG signal was undetectable for the annealed sample probably due to the introduction of extrinsic defects. This result confirms that non-centrosymmetry and ferroelectricity are very sensitive to the crystalline quality. The scale bars represent 500 nm.

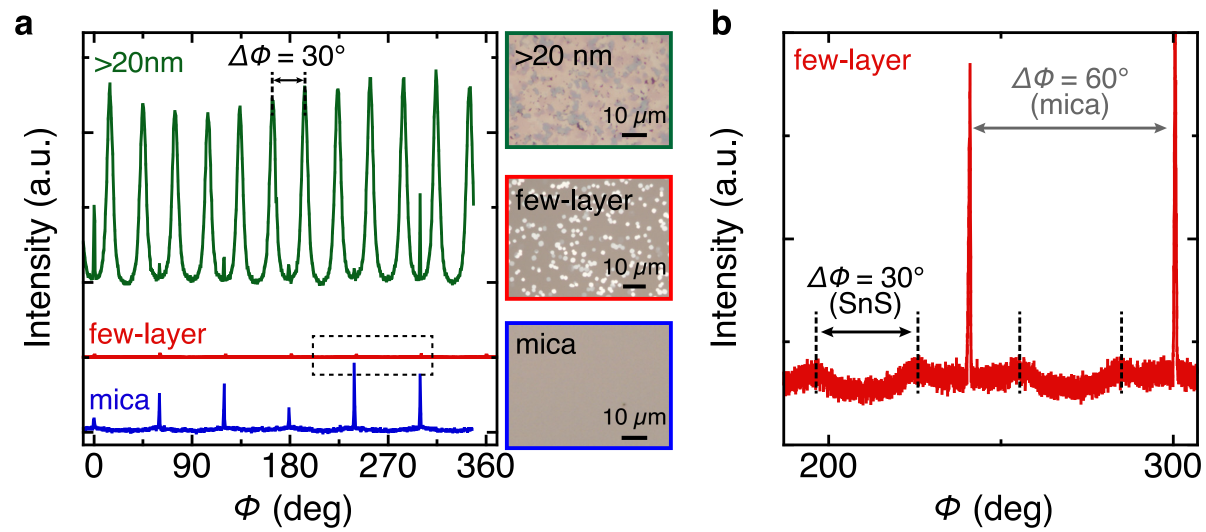

Supplementary Fig. 5 XRD analysis of SnS grown on mica. **a** In-plane XRD Φ-scan for PVD grown SnS on mica measured with the samples tilted at an angle of $\chi = 23.3°$, which corresponds to the angle between SnS(016) and (001) planes[1]. The growth time was changed from 2 to 15 min. The 15-min grown sample was full covered with >20 nm SnS crystals, whereas the 2-min grown sample has small coverage with isolated multi-layer SnS crystals. As a reference, the Φ-scan of intrinsic mica substrate is shown at the bottom. For mica, six peaks were observed with an interval of 60°. The fluctuation of diffraction intensity is probably owing to an inhomogeneous distribution of SnS crystals on mica substrate. **b** Φ-scan for few-layer SnS with the selected region as shown in **a**. The Φ-scan of SnS(016) plane indicates 12 peaks with an interval of 30° for few-layer and >20 nm SnS, which agrees well with the reported data for SnS on mica[1]. These results indicate a strong interaction between SnS and mica probably due to a lattice matching. Although the crystallographic interaction between SnS/mica may disappear with increasing the SnS thickness, there is still a preferred orientation for SnS growth as long as the thickness is several-tens nanometers.

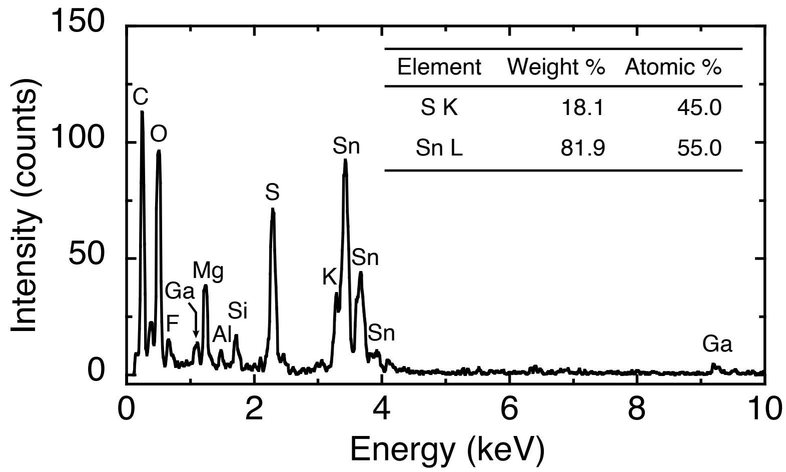

Supplementary Fig. 6 Typical cross-sectional EDS spectrum of trilayer SnS.

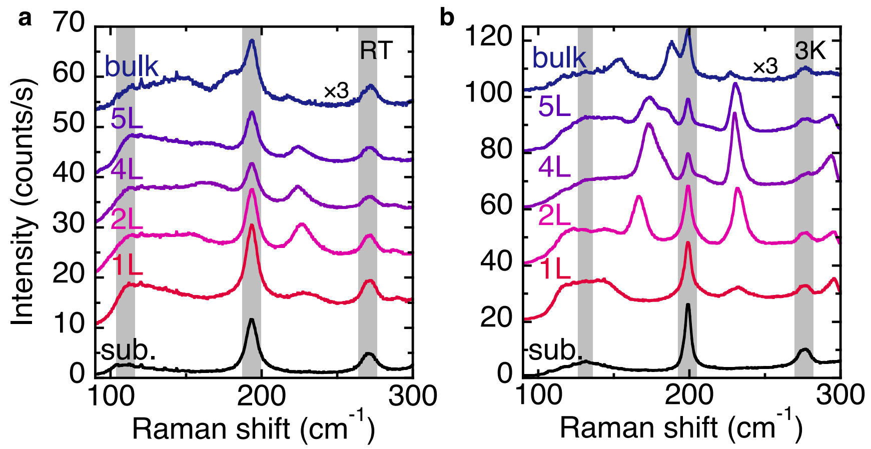

Supplementary Fig. 7 Typical Raman spectra for SnS with different thicknesses from bulk to monolayer, measured at **a** RT and **b** 3K.

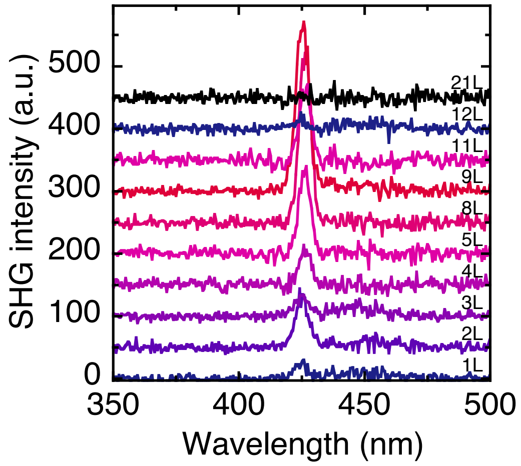

Supplementary Fig. 8 Typical RT SHG spectra for SnS with different thicknesses.

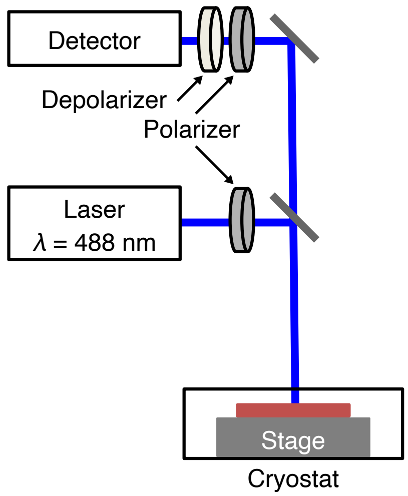

Supplementary Fig. 9 Schematic diagram of polarized Raman spectroscopy. The polarization angle was changed through rotating the polarizers. The second polarizer was set to be parallel or perpendicular to the polarized incident light for Raman measurement.

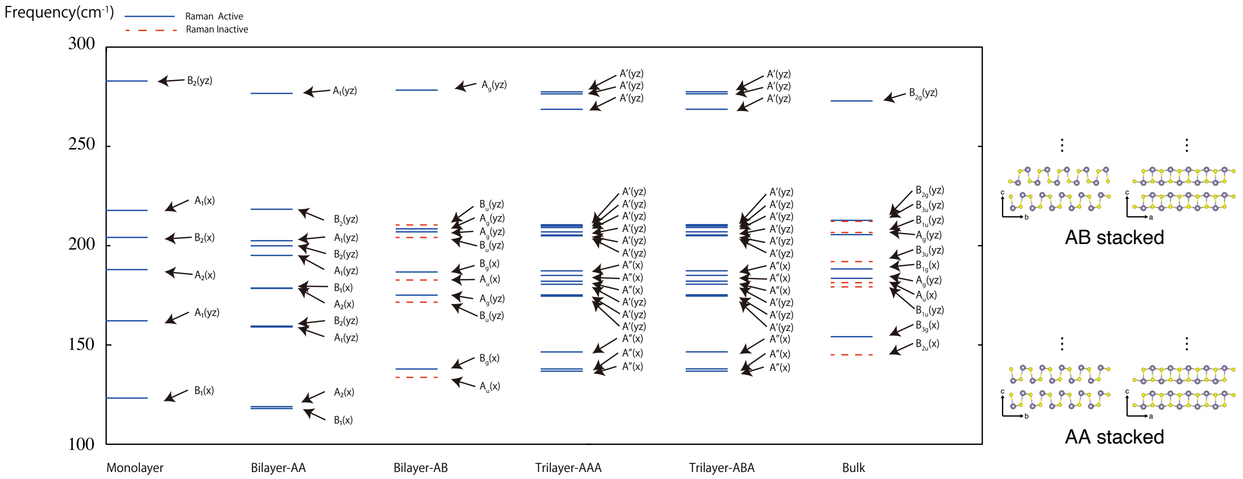

Pmn2_1(31) ··· Mono,Bilayer-AA	P2_1/m(11) ··· Bilayer-AB	Pm(6) ··· Trilayer-AAA,Trilayer-ABA	Pnma(62) ··· Bulk
mm2 , C_{2v}	2/m , C_{2h}	m , C_s	2/mmm , D_{2h}
A_1···z,x^2,y^2,z^2	A_g···R_z,x^2,y^2,z^2,xy	A′···x,y,R_z,x^2,y^2,z^2,xy	A_g···x^2,y^2,z^2
A_2···R_z,xy	B_g···R_x,R_y,yz,zx	A″···z,R_x,R_y,yz,zx	B_{1g}···R_z,xy
B_1···x,R_y,zx	A_u···z		B_{2g}···R_y,zx
B_2···y,R_x,yz	B_u···x,y		B_{3g}···R_x,yz
			A_u···nothing
			B_{1u}···z
			B_{2u}···y
			B_{3u}···x

Supplementary Fig. 10 Calculated Raman active/inactive phonon modes for monolayer, AA/AB-bilayer, AA/AB-trilayer, and AB-bulk SnS. The point group, irreducible representation of phonon modes, and vibration direction are summarized.

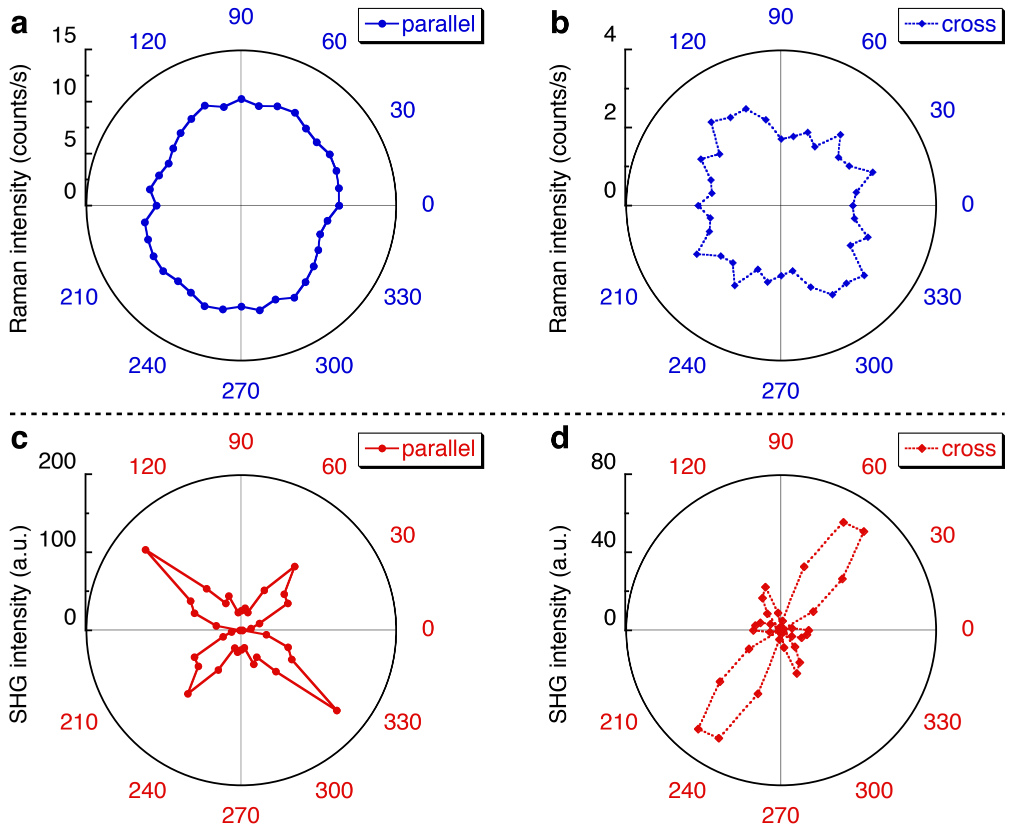

Supplementary Fig. 11 Angular dependence of optical measurements for 10L SnS: Raman intensity at ~226 cm^{-1} under **a** parallel and **b** perpendicular polarization, and SHG intensity under **c** parallel and **d** perpendicular polarization. Note that different 10L SnS samples were used for each measurement. A strong anisotropy was confirmed for the SHG intensity, though angular dependence were slight for the Raman measurements. This discrepancy is probably due to the difference of crystalline quality, which also resulted in the large distribution of SHG intensity even for the same thickness (**Fig. 5b**).

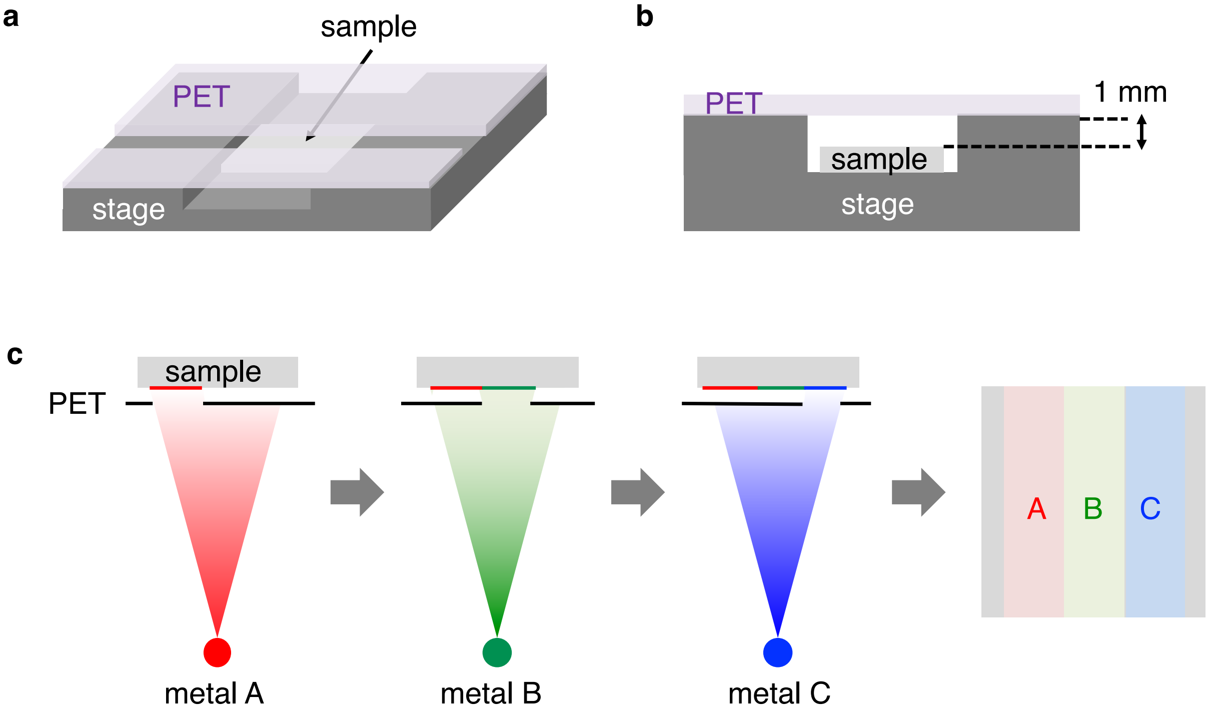

Supplementary Fig. 12 Fabrication method of multiple metal depositions. **a** Bird's-eye view and **b** cross-sectional view of sample stage with PET shield. **c** Step-by-step deposition of multiple metals.

Method: After the EB lithography for the electrodes, the sample was set on a home-made stage with hollow (**a,b**). The sample surface was partially covered with the PET shield, which was placed approximately 1 mm apart from the sample. Each metal was deposited step-by-step with changing the position of the PET shield by using a standard thermal evaporator (**c**). Finally, SnS devices with different electrode metals were obtained on the same wafer, as shown in Fig. 4a.

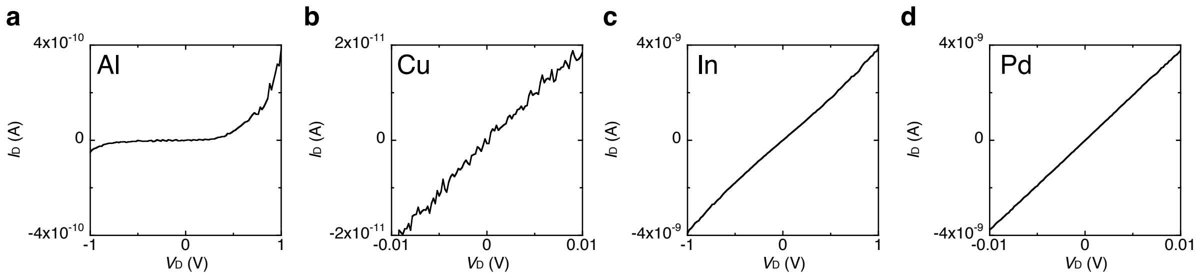

Supplementary Fig. 13 Typical I_D–V_D curves for bulk SnS with different contact metals: **a** Al, **b** Cu, **c** In, and **d** Pd.

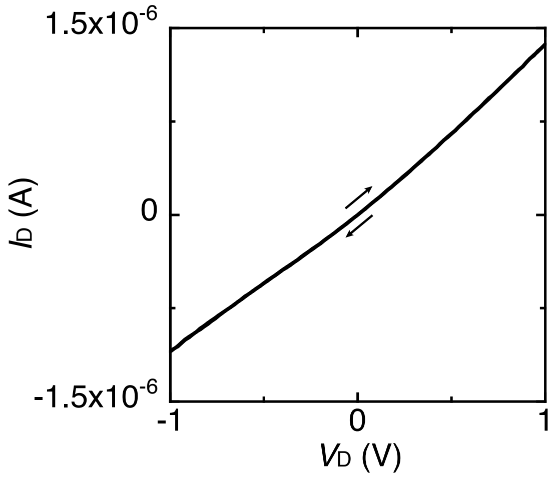

Supplementary Fig. 14 Typical I_D–V_D curve with Ni contact for few-layer SnS below the critical thickness for ferroelectricity.

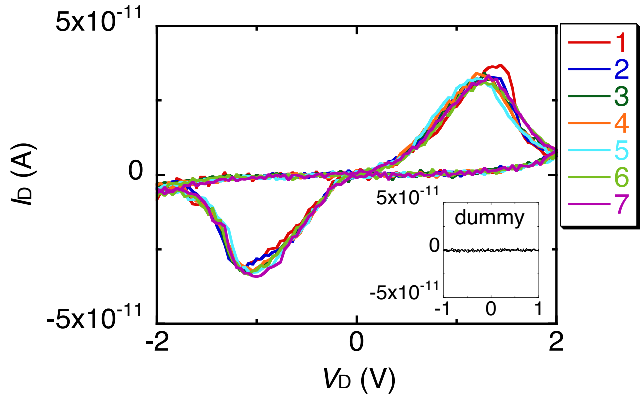

Supplementary Fig. 15 Ferroelectric switching characteristic of I_D–V_D for Ag/9L-SnS cycled 7 times at RT. Inset: I_D–V_D for a dummy sample without SnS. Ferroelectric hysteresis was absent for the dummy device without SnS. The reproducible switching characteristic was observed in the multiple measurements, indicating the stability of ferroelectricity.

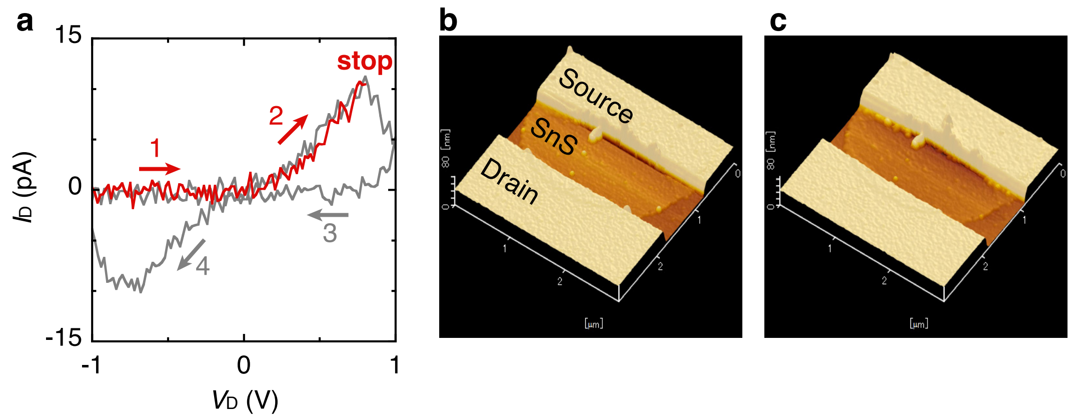

Supplementary Fig. 16 Exclusion of Ag diffusion into SnS. **a** I_D–V_D curves for 2.6 nm (~4L) SnS with Ag electrodes. A single sweep from −1 V to 800 mV stopped at the low resistive state is shown in a red line. For comparison, a double sweep is shown in a gray line. AFM topographic images **a** before and **b** after applying the external electric field. The scale bars represent 500 nm.

AFM topographic image was observed after the I_D–V_D measurement. In order to observe the SnS surface at the low resistive state (LRS), the drain voltage was swept from −1 V to 800 mV just before the AFM measurement (a). No significant Ag diffusion was confirmed in the channel region at the LRS compared with the AFM image before the electrical measurement (b,c).

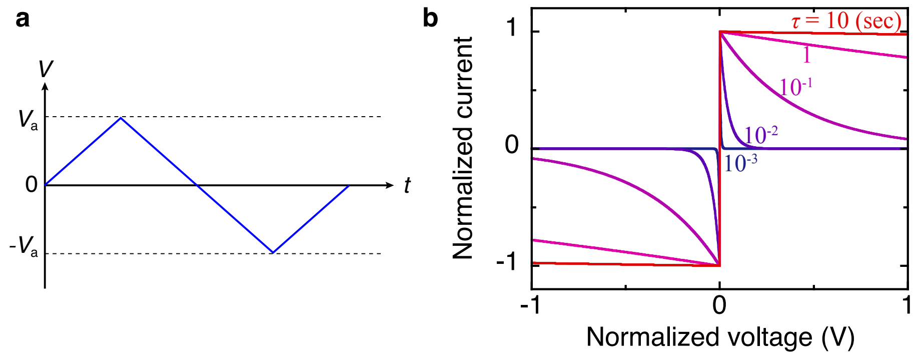

Supplementary Fig. 17 Theoretical analysis of *I*–*V* hysteresis loop based on the carrier trapping model. **a** The schematic diagram of applied voltage. **b** Calculated relationship between voltage and current from carrier emission. The emission time constant τ was changed from 1 ms to 10 s.

Analysis: False ferroelectricity in nonferroelectric systems has been theoretically investigated based on the carrier trapping model[2]. In this model, the following properties are assumed; 1) the sample is a semiconductor with Schottky contacts on both the source and drain contacts, 2) a large concentration of trap sites is located over a finite thickness w_t at the semiconductor/metal interfaces, 3) the normal leakage current is neglected and the carrier emission from the traps is dominant, and 4) the displacement current due to the space charge near the contacts produced by the trapping/detrapping is neglected. Based on this model, the carrier emission from the traps I_{tr} can be written as

$$I_{tr} = \frac{qAw_tN}{\tau} \exp\left(-\frac{V}{4\tau V_a f}\right) \quad \text{(S1)}$$

where A is the area of the electrode, N is the initial concentration of occupied traps, τ is the emission time constant from the traps, V is the applied source–drain voltage, V_a is the amplitude of the applied signal (Supplementary Fig. 13a), and f is its frequency.

To exclude the possibility that the present ferroelectric switching behavior is due to the carrier emission from the traps, we discuss the suitability of carrier trapping models assuming that the present device is not ferroelectric. When the relationship of current and voltage is calculated based on Eq. (S1), the shape of the I–V curve strongly depends on τ, as shown in Supplementary Fig. 13b. Comparing the I–V shapes of the calculated and experimental results (Figs. 4c,e and 5a), the value of τ is approximately estimated to be in the order of 100 ms. The maximum current I_{max} can be obtained when the voltage is close to zero. Substituting $V = 0$ V into Eq. (S1), the total number of traps Aw_tN can be expressed as follows,

$$Aw_tN = \frac{\tau}{q} I_{max} \quad \text{(S2)}$$

From the experimental results, I_{max} is ranged up to 150 pA (Fig. 4e). When we substitute $\tau = 100$ ms and $I_{max} = 150$ pA, the total number of traps is determined to be 9×10^7 states near the Ag/SnS interface in the SnS channel (the channel thickness, length, and width are 5.4 nm, 0.4 µm, and 3.0 µm, respectively). In this case, the number of traps corresponds to one in three of the total number of atoms in the SnS channel, which is too large intuitively. From these results, the effect of carrier emission from the traps can be neglected for the present ferroelectric I–V hysteresis loops.

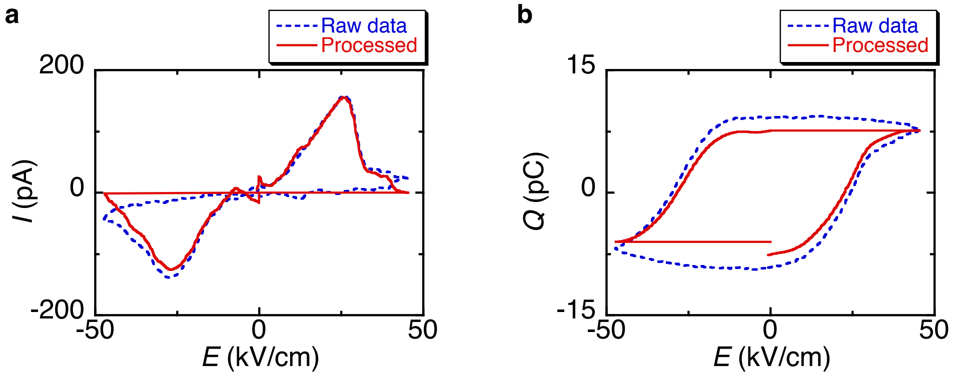

Supplementary Fig. 18 Background subtraction for I–E and Q–E hysteresis loop. **a** To eliminate the residual contribution of leakage current in I–E hysteresis loop, the current value at the high resistive state was subtracted from that at the low resistive state. **b** Q–E hysteresis loop with and without the background subtraction.

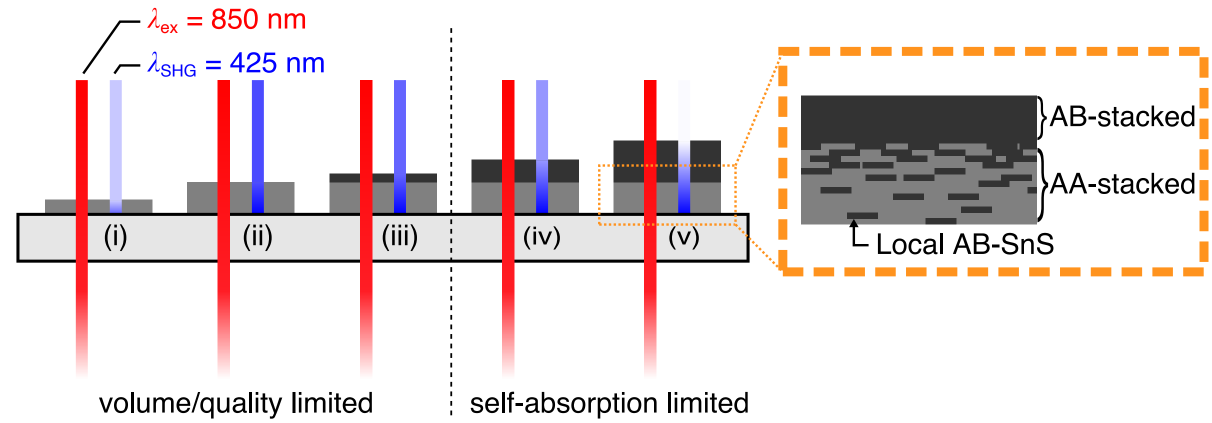

Supplementary Fig. 19 Schematic illustration of a model for interlayer coupling in multilayer SnS. The red and blue lines indicate the excitation laser (λ = 850 nm) and SHG signal (λ = 425 nm), respectively.

In this model, for the thin SnS crystals, the stacking sequence is dominated by AA stacking probably due to the effect of strain introduced through the interaction with the mica substrate. In contrast, above the critical thickness of ~15L, the stacking sequence is gradually changed to AB stacking, which is a thermodynamically stable state. In the SHG measurements, an 850-nm excitation laser, whose penetration depth in SnS is at least of several-hundred-nm[3,4], was used to generate the SH signals. Thus, in the case of SnS thinner than ~20L as discussed in this work, the whole region is excited. When the SnS thickness reaches the critical thickness at (iii), the SHG signal is maximized. Above the critical thickness, although the volume of non-centrosymmetric region does not change, the self-absorption of the SHG signal at 425 nm becomes effective because its penetration depth is much shorter than 850 nm[3,4]. As a result, the SHG intensity decreases with increasing SnS thickness and is finally annihilated.

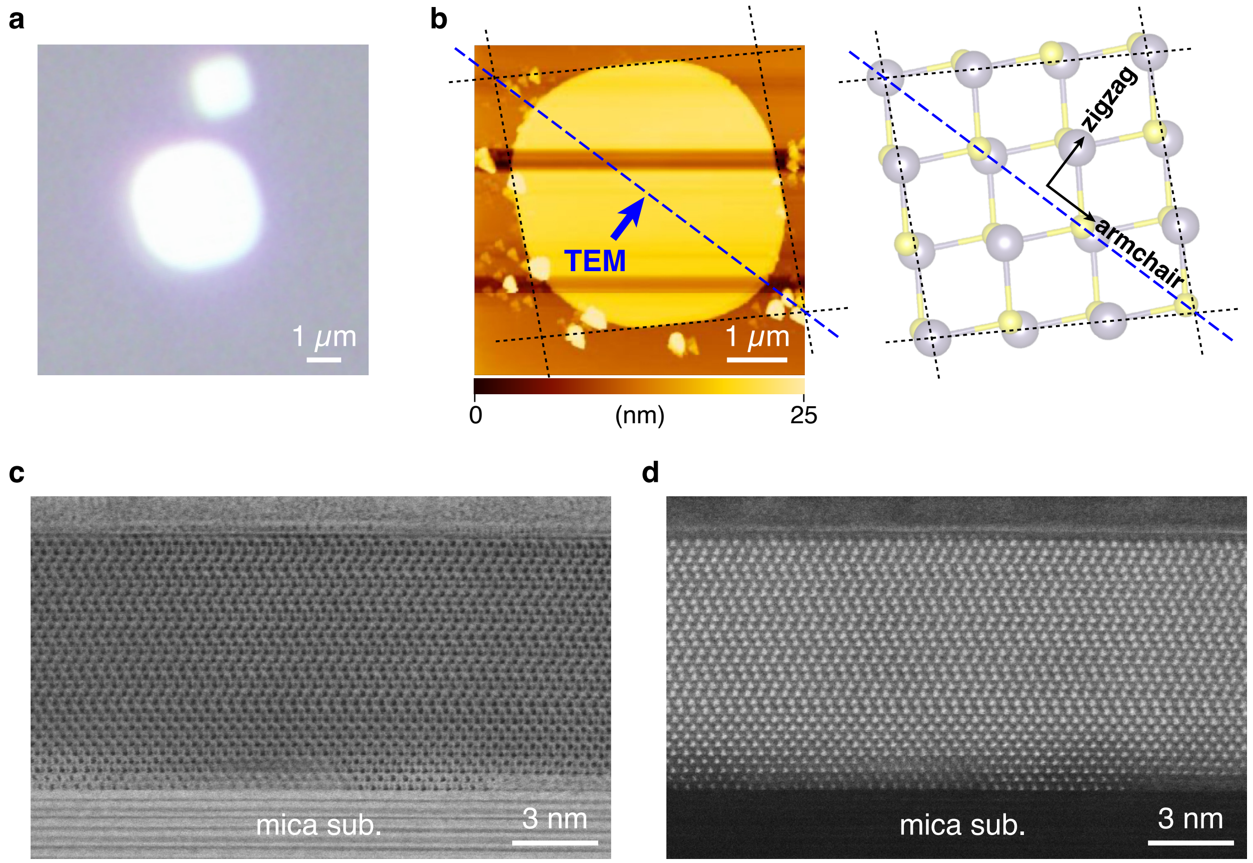

Supplementary Fig. 20 Cross-sectional TEM observation of PVD grown SnS on mica. **a** Optical and **b** AFM topographic images of 16L SnS. The sample was cut along the armchair direction, as shown in a dashed blue line. **c** Bright-field STEM and **d** HAADF-STEM images of 16L SnS.

Supplementary References

1. Wang, S. F., Fong, W. K., Wang, W. & Surya, C. Growth of highly textured SnS on mica using an SnSe buffer layer. *Thin Solid Films* **564**, 206–212 (2014).
2. Pintilie, L. & Alexe, M. Ferroelectric-like hysteresis loop in nonferroelectric systems. *Appl. Phys. Lett.* **87**, 112903 (2005).
3. Banai, R. E. *et al.* Optical Properties of Sputtered SnS Thin Films for Photovoltaic Absorbers. *IEEE J. Photovoltaics* **3**, 1084–1089 (2013).
4. El-Nahass, M. M., Zeyada, H. M., Aziz, M. S. & El-Ghamaz, N. A. Optical properties of thermally evaporated SnS thin films. *Opt. Mater.* **20**, 159–170 (2002).
